# Correlation of choroidal thickness and ametropiain young adolescence

**DOI:** 10.1371/journal.pone.0174385

**Published:** 2017-04-12

**Authors:** Xiaolei Shao, Chang Zou, Bo Qin

**Affiliations:** 1 Shenzhen Eye Hospital, Affiliated Shenzhen Eye Hospital of Jinan University, Joint College of Optometry, Shenzhen Universtiy, Shenzhen Key Laboratory of Ophthalmology, Ocular Trauma Treatment and Stem Cell Differentiation Public Service Platform of Shenzhen, Guangdong Province, China; 2 Clinical Medical Research Center, The Second Clinical College of Jinan University, Shenzhen People’s Hospital, Shenzhen, China; Bascom Palmer Eye Institute, UNITED STATES

## Abstract

Choroid has been proposed to participate in the regulation of light refraction by changing its thickness. The present study aims to analyze the characteristics of choroidal thickness (CT), and its correlation with refractive error, axial length and age in young ametropia. A total of 51 subjects (102 eyes), aged from 5 to 18 years old (mean age 10.04 ±2.78 years), with ametropia were included in the study. Choroidal imaging was obtained by enhanced depth imaging (EDI) of spectral domain Optical Coherence Tomography (OCT). CT was horizontally measured at 5 locations in across fovea with 1mm interval. We found that the spherical equivalent refractive diopter was from -7.25D to 1.6D (mean, -1.61D±1.82D), the mean axial length was 24.14mm±1.14mm. The closer to the optic disc the thinner the choroid is. CT between fovea and disc showed better correlation with refractive error (p< 0,01), axial length (p<0.01) and age (P<0.05) than those temporal to fovea. Our results indicated that the choroid is least thick around the optic disc. Thickness between fovea and optic disc is significantly associated with refractive error, axial length and age in growing adolescences. This result may help us understand the function of choroid during ametropic progression.

## Introduction

Choroid is the posterior part of uvea, which is rich of blood vessels and pigment. It supplies oxygen and nutrients to the outer retina [1]. Abnormality of choroid is closely related to fundus diseases, such as polypoidal choroidal vasculopathy, exudative age-related macular degeneration[2], uveitis [3] and high axial myopia [4]. Although choroid is not involved in the visual signal conduction, it has been proposed to participate in the regulation of light refraction [5]. Previous study indicated that choroid might regulate light refraction by changing its thickness in chicken’s eye[6].

Atrophy and bleeding of macular and peripapilary choroid in high myopic eye occurs with high frequencies in clinical practice. It is reported that the thickness of choroid in patients with high myopia is thinner than that in normal eye[7].Refractive error, axial length and age have been reported to be associated with choroidal thickness in healthy adult population [8–10]. The human eyes are born in a hyperopic status. The axial length grows along with increasing age and stops growing at about 18 to 20 years old. Many people suffer from ametropia when their eyes undergo the process of emmetropization. The exact function of choroid in the development of ametropia remains unclear.

The enhanced depth imaging (EDI) modality of Optical Coherence Tomography (OCT) enables the non-invasive measurement of choroidal thickness in vivo[8, 11–15].In this study, we aim to accumulate the preliminary evidences for deciphering the function of choroid thickness in the formation of ametropia. A total of 51 young subjects with myopia or hyperopia were observed. Choroidal thickness at 5 locations on the central horizontal scan was measured and their association with axial length, refractive error and age was analyzed.

## Subjects and methods

### Study subjects

We randomly selected adolescents aged from 5 to 18 years who visited the optometry clinic of Shenzhen Eye hospital from Nov 2014 to Dec 2014. A total of 51 subjects whose uncorrected visual acuity less than 20/25were included in the study. The exclusion criteria included: 1) Subjects who has the history of any other eye diseases, such as cataract, glaucoma, strabismus et al. 2) The best corrected visual acuity is less than 20/25 or the cylinder lens more than 2D. 3) The intraocular pressure (IOP) is out of the normal range (11-21mmHg) and ratio of the optic nerve cup to optic disc is>0.5, or binocular asymmetry >0.2. 4) Subjects with the history of any ocular trauma or surgery. 5) Subjects with any history of systematic diseases. 6) The inner and outer boundary of choroid cannot be clearly detected on the OCT picture.

The study protocol had been approved by the Ethics Committee of Shenzhen Eye Hospital. The research adhered to the Declaration of Helsinki. We obtained written informed consent from the guardians on behalf of the children enrolled in our study.

### Ophthalmologic examination

Every studied subject received a comprehensive ophthalmologic test by an experienced ophthalmologist. Examinations included the uncorrected visual acuity test with Snellen chart, motility test, intraocular pressure test, slit lamp exam and dilated fundoscopy examination.

### Cycloplegic refraction

For the subjects under 12 years old, pupils were dilated using 1% atropine ointment for 3 days. For the subjects over 12 years old, pupils were fast dilated using Compound Tropicamide Eye Drops (Santa, Japan). All the refraction examinations were performedby the same experienced optometrist. Refractive error (RE) was recorded as spherical equivalence for statistical analysis. The degree of spherical equivalence was calculated as Degree of Sphere +1/2 Degree of Cylinder.

### Choroidal thickness measurement

**Fig 1:**
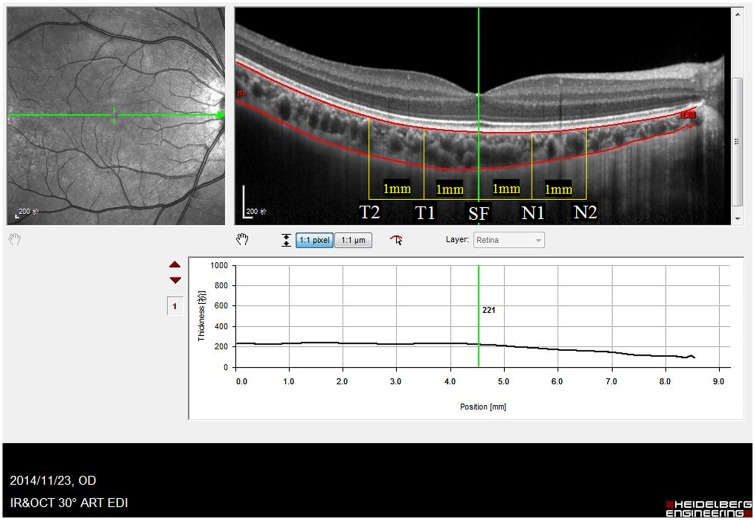
Choroidal thickness measurement. The cross-sectional image of choroid was obtained in Enhanced Depth Imaging (EDI) scan of Optical Coherence Tomography. The horizontal scan line is centered on fovea. The outer border of RPE and the outer border of choroid were bounded by two lines. The choroidal thickness was measured as the distance between the two boundary lines. Five locations with 1mm interval (T2,T1,SF,N1,N2)were measured. The sample image was from the right eye of a ten-year-old boy with -3.0D refractive error. T2 = 2mm temporal to fovea, T1 = 1mm temporal to fovea, SF = subfovea, N1 = 1mm nasal to fovea, N2 = 2mm nasal to fovea.

The choroidal thickness was measured by EDI mode of spectral domain OCT (SPECTRALIS HRA+OCT, Heidelberg, Germany). The horizontal single line scan through fovea was performed on each eye. We used "thickness profile" mode of the SPECTRALIS system. Since the system does not have the automatic choroidal thickness analysis, so we chose the "retina" mode. The system automatically drew the borderline of the retina when "retina" was chosen. We kept the outer borderline of the retina and define it as the inner border of the choroid, and then move the inner borderline of the retina to the outer border of choroid manually, then move the green line at each test position and get the thickness number from the lower chart. The choroidal thickness is defined as the distance between the outer border of retina pigment epithelium (RPE) and the sclera-choroidal interface (see in Fig 1). Five locations with 1mm interval on the scan line were selected to measure the choroidal thickness: subfovea (SF), 1mm nasal to fovea (N1), 2mm nasal to fovea (N2), 1mm temporal to fovea (T1) and 2mm temporal to fovea (T2). All the OCT examinations were performed under non-mydriatic conditions. All the OCT scans were analyzed by one observer. The observer was blinded with the refraction and axial length information of patients when analyzingthe choroidal thickness. We had considered the possible fluctuation of the choroidal thickness at different time during a day and performed all the examination in the morning from 9-11am.

### Axial Length (AL) measurement

IOL master (Zeiss, Germany) was used to measure the axial length of each eye. Multiple AL measurements (at least 5) were taken and the average value was recorded.

### Statistical analysis

The paired sample T-test was utilized to analyze the differences in choroidal thickness between neighboring locations, right and left eyes, male and female genders. Linear regression and Pearson correlation were calculated for variance in choroidal thickness relative to refractive error, axial length and age. The correlation coefficient was reported according to the Pearson correlation factor, R^2^ and P value. P value <0.05 was considered as of statistical significance.

## Results

There were 51 subjects participated in this research, including 26 boys and 25 girls. The mean age was 10.04±2.78 years (ranged from 5 to 18 years). The mean of spherical equivalent refractive error was -1.61D±1.82D (ranged from -7.25D to 1.6D). The average axial length was 24.14±1.14 mm (ranged from 21.9mm to 27.4 mm). Original measured data of all the subjects was listed in S1 File. There was no statistical significance of spherical equivalent refractive error or axial length between right and left eyes (p>0.05) in these subjects.

On the horizontal central scan line, the choroidal thicknesses (CT) decreased from temporal to nasal. In the five locations, T2 was the thickest, while N2 was the thinnest point. Details were shown in Table 1. The differences between CT of neighboring locations were analyzed by using paired sample T-test. Statistical significances were detected in two neighboring pairs: N2 and N1 (p = 0.000), N1 and SF (P = 0.000). There were no significant difference between T1 and T2, T1 and SF. There was no statistical significance between the CT of right eye and left eye (p>0.05).

**Table 1:** Choroidal Thickness (CT) of each measurement location.

Measurement location	CT (Mean±SD)
2mm temporal to fovea (T2)	296.85±58.90μm
1mm temporal to fovea (T1)	292.14±62.94μm
subfovea (SF)	287.42±70.37μm
1mm nasal to fovea (N1)	247.34±64.70μm
2mm nasal to fovea (N2)	201.08±58.80μm

**Fig 2:**
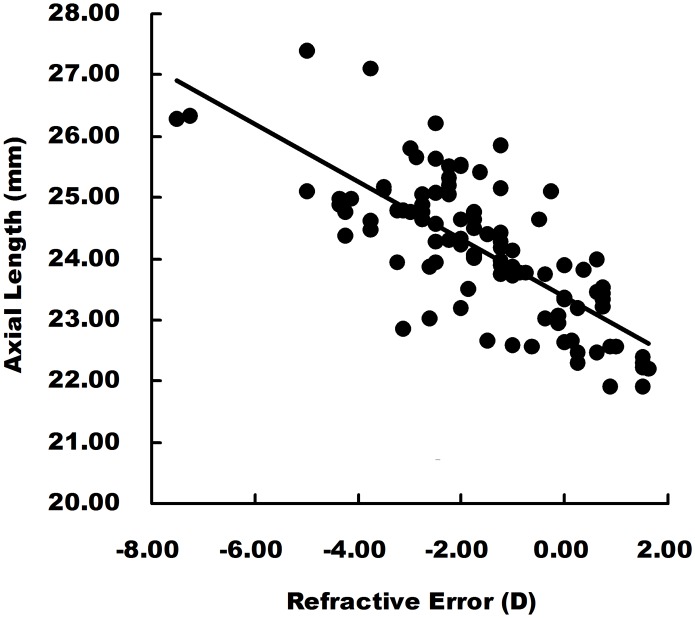
Linear regression analysis of axial length and refractive error. Refractive error showed significantly negative correlation with axial length (R^2^ = 0.5616, F = 128.023 p = 0.000), linear equation: y = -0.472x + 23.382.

In the correlation analysis, the refractive error (RE) was negatively associated with the axial length (AL) (R^2^ = 0.5616, F = 128.023, p = 0.000). Scatter plot and linear regression were shown in Fig 2. The choroidal thicknesses of all the locations were positively associated with RE (p<0.05) and negatively associated with AL (p<0.05); The CT on N1 and SF were negatively correlated with age (p = 0.01 and p = 0.007). However, no statistical significance was detected on the other three locations (p>0.05). Correlation analysis results on CT were listed on Table 2.

**Table 2:** Correlation analysis between choroidal thickness of each point and Refractive Error (RE), Axial Length(AL) and age.

	Correlation with RE			Correlation with AL			Correlation with age		
	Pearson correlation	R^2^	p value	Pearson correlation	R^2^	p value	Pearson correlation	R^2^	p value
CT_N2_	0.411	0.169	0.000	-0.387	0.150	0.000	-0.137	0.019	0.171
CT_N1_	0.489	0.239	0.000	-0.424	0.180	0.000	-0.263	0.069	0.007
CT_SF_	0.481	0.231	0.000	-0.421	0.177	0.000	-0.255	0.065	0.01
CT_T1_	0.389	0.151	0.000	-0.308	0.095	0.002	-0.182	0.033	0.069
CT_T2_	0.312	0.097	0.001	-0.203	0.041	0.041	-0.152	0.023	0.127

RE = refractive error; AL: axial length; CT = choroidal thickness; N2 = 2mm nasal to fovea;

N1 = 1mm nasal to fovea; SF: subfovea; T1 = 1mm temporal to fovea; T2 = 2mm temporal to fovea

p<0.05 indicates statistical significance.

Although the axial length increased along with age (R^2^ = 0.341, p = 0.000), only the CT in SF and N1 showed significant association with age. In the multiple regression analysis, when age was added into the regression between CT and AL, the change of R^2^ was negligible (Table 3).

**Table 3:** Multiple regression of CT(sf and n1) with age and AL.

			multiple R	R^2^	R^2^ change	F	P
CT_SF_	step 1	AL	0.42076	0.17704	0.17704	21.51287	0.00001
	step 2	AGE	0.42076	0.17704	-0.00013	0.01617	0.89906
CT_N1_	step 1	AL	0.42417	0.17992	0.17992	21.93929	0.00001
	step 2	AGE	0.42417	0.17992	-0.00038	0.04555	0.83143

RE = refractive error; AL: axial length; CT = choroidal thickness; N1 = 1mm nasal to fovea; SF: subfovea; p<0.05 indicates statistical significance.

**Fig 3:**
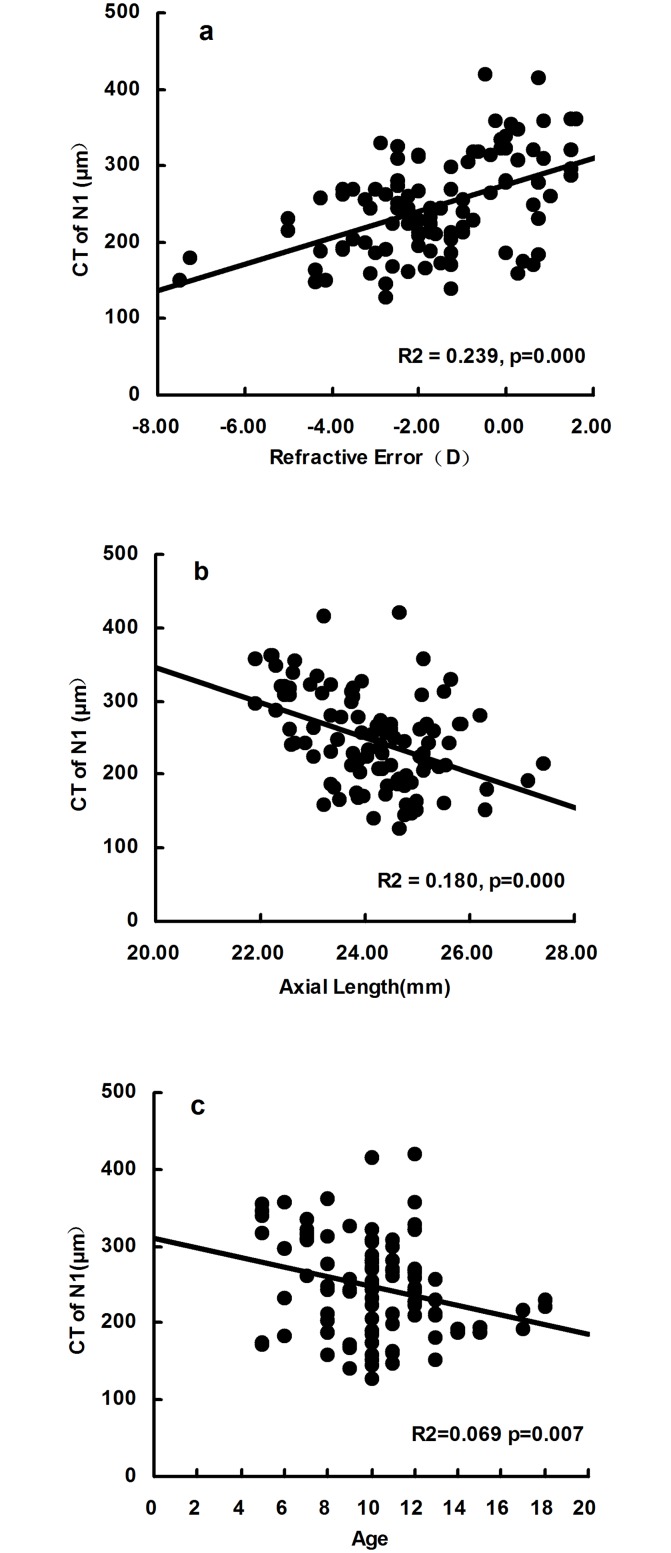
The scatter plot graphs showing significant correlation between Choroidal Thickness(CT) of N1 and three factors (refractive error, axial length and age). **a**, CT of N1 negatively correlated with refractive error (diopter) (R^2^ = 0.239, p = 0.000).**b**, CT of N1 positively correlated with axial length (R^2^ = 0.180, p = 0.000).**c**, CT of N1 positively correlated with age (R^2^ = 0.069, p = 0.007). N1 = 1mm nasal to fovea.

The CT on N1 demonstrated best association with the RE (R^2^ = 0.239, p = 0.000), AL (R^2^ = 0.180, p = 0.000) and age (R^2^ = 0.069, p = 0.007) among all 5 measurement points. The scatter plot graphs were shown in Fig 3.

## Discussion

Emmetropization is a vision dependent mechanism that attempts to minimize the refractive error through coordinated growth of eyeball, such that axial length matches the focal length [16]. This kind of vision guided eye growth results in a scleral remodeling in response to visual stimuli characterized by changes in proteoglycan synthesis, collagen synthesis, and matrix metalloproteinase activity[17–19]. Choroid plays an essential role in emmetropization[16]. In addition to directly release growth factors to regulate scleral proteoglycan synthesis, choroid also play an accommodation function by modulating its thickness[1, 6]. Studies on chicken eyes showed that the thickness of choroid increased and move the retina forward to compensate the myopic defocus, or decreased and move the retina backward in the case of hyperopic defocus [1, 6]. In the research on human eyes, the thickness of choroid decreased during the hyperopic accommodation [20].On the other hand, choroid might also modulate the sclera growth by passing through the molecular signals [21].

It is reported that the choroidal thickness is not equal around the fundus. Rossue et al proved that the choroidal thickness increased as the distance from the optic disc increased[13]. The increase is more obvious in high myopic eyes. Ho et al demonstrated that the point closest to optic nerve had thinnest choroidal thickness among all the measure points [11]. These findings were identified in our present study. It was observed that the CT reduced from the temporal point to the nasal point on the central horizontal scan line. From fovea to optic disc, the thinning of choroid became more and more remarkable. The changes of CT between adjacent points were statistically significant.

In the present study, we found that the choroidal thickness is negatively associated with axial length and positively associated with refractive error. According to the linear regression, the subfoveal CT lost 25.891 μm thicknesses for each millimeter axial elongation and 18.641 μm for each diopter refractive error reduction. This finding coincides with previous observation that CT decreased by 9.3 μm to 15 μm for every decrease in refractive dioper of 1 D [8, 9, 22]. CT reduced by 22μm to 32μm for every increase in axial length of 1mm [9, 22]. We also found that correlation coefficients varied in different points when correlated choroidal thickness with axial length and refractive error. The choroidal thickness showed better association with axial length and refractive error at the points between fovea and disc when compared with those temporal to fovea. This finding indicated that the choroidal thickness between fovea and disc is more sensitive to the changeof axial length and refractive error. This might explain why high myopic atrophic change started and mainly happened at this posterior region. The choroid of this region might play an important role during the development of ametropia.

Controversial findings were reported in the correlation between subfoveal CT and age[8–11, 23, 24]. In those studies, there were large age span of the studied subjects that might contribute to the conclusion bias. Considering the development of visual axis is age related, particularly in adolescences, the present study recruited subjects that are the growing adolescences with refractive error. Significant negative correlation between choroidal thickness and age at location SF and N1 were found in this study, which indicates that choroidal thickness of the posterior pole decreased as age increased.

This and other study demonstrated that the choroid participates the refraction process by changing its thickness. Also, choroid might have essential correlation with the pathological change of eyes. In high myopic patients, choroidal atrophy can often be detected at the posterior pole around optic nerve and macula. As the major blood supplier of retina, especially for the outer layer, the choroidal thickness may directly reflect the nourishment of photoreceptors, and therefore will affect the visual acuity. Some researchers proved that the subfoveal choroidal thickness is positively correlated with the visual acuity in high myopic patients [11, 25]. The choroidal thickness between disc and fovea might be a good predictor of visual outcome in myopic patients.

Of note, the EDI-OCT was scanned for two high hyperopic patients. Their data were excluded because of low corrected visual acuity and un-displayable sclera expression in OCT image. Their refractive diopters were from +5.5D to +7.0D, and their CT was over 500 μm. Since the sclera reflection cannot be detected in the image, their CT was immeasurable. Therefore, the penetration of the 870 nm laser used in EDI-OCT is still limited. When the choroid is thicker than 500 μm, it will be hard to reach sclera. Recently, high penetrating OCT (HP-OCT) by using longer wavelength light (1050-1060nm) began to be used in clinical practice with better visibility of chorioscleral interface than SD-OCT [26]. This new type of OCT might be a better choice for measuring choroidal thickness in high hyperopic patients with much thicker choroid.

The limitation of this study is the small sample size. In order to increase the sample number in analysis, we included both eyes of the sample into the study. This might introduce selection bias. A study with large sample size will be performed in the future to further confirm the results. In addition, all the analysis on choroidal thickness was performed by one observer, it might introduce some bias too.

In conclusion, the choroid thickness around the optic disc and macula is significantly associated with refractive error, axial length and age in growing adolescences. This resultmay help us understand the function of choroid during ametropic progression. It also provides comparative value for future research on the pathophysiological process of myopic progression.

## Supporting information

S1 File Original data. Original measured data and personal information of subjects.
(XLSX)

## References

[1] Nickla DL, Wallman J. The multifunctional choroid. Progress in retinal and eye research 2010;29:144–68. 10.1016/j.preteyeres.2009.12.002 20044062 PMC2913695

[2] Chung SE, Kang SW, Lee JH, Kim YT. Choroidal thickness in polypoidal choroidal vasculopathy and exudative age-related macular degeneration. Ophthalmology 2011;118:840–5. 10.1016/j.ophtha.2010.09.012 21211846

[3] Gehl Z, Kulcsar K, Kiss HJ, Nemeth J, Maneschg OA, Resch MD. Retinal and choroidal thickness measurements using spectral domain optical coherence tomography in anterior and intermediate uveitis. BMC ophthalmology 2014;14:103 10.1186/1471-2415-14-103 25176513 PMC4236668

[4] Jonas JB, Xu L. Histological changes of high axial myopia. Eye 2014;28:113–7. 10.1038/eye.2013.223 24113300 PMC3930258

[5] Walls GL. The vertebrate eye and its adaptive radiation. Bloomfield Hills, Mich.,: Cranbrook Institute of Science; 1942.

[6] Wildsoet C, Wallman J. Choroidal and scleral mechanisms of compensation for spectacle lenses in chicks. Vision research 1995;35:1175–94. 7610579 10.1016/0042-6989(94)00233-c

[7] Wang S, Wang Y, Gao X, Qian N, Zhuo Y. Choroidal thickness and high myopia: a cross-sectional study and meta-analysis. BMC ophthalmology 2015;15:70 10.1186/s12886-015-0059-2 26138613 PMC4490603

[8] Ding X, Li J, Zeng J, Ma W, Liu R, Li T, et al Choroidal thickness in healthy Chinese subjects. Investigative ophthalmology & visual science 2011;52:9555–60. 22058342 10.1167/iovs.11-8076

[9] Ikuno Y, Kawaguchi K, Nouchi T, Yasuno Y. Choroidal thickness in healthy Japanese subjects. Investigative ophthalmology & visual science 2010;51:2173–6. 19892874 10.1167/iovs.09-4383

[10] Chhablani J, Rao PS, Venkata A, Rao HL, Rao BS, Kumar U, et al Choroidal thickness profi le in healthy Indian subjects. Indian journal of ophthalmology 2014;62:1060–3. 10.4103/0301-4738.146711 25494246 PMC4290194

[11] Ho M, Liu DT, Chan VC, Lam DS. Choroidal thickness measurement in myopic eyes by enhanced depth optical coherence tomography. Ophthalmology 2013;120:1909–14. 10.1016/j.ophtha.2013.02.005 23683921

[12] Fujiwara A, Shiragami C, Shirakata Y, Manabe S, Izumibata S, Shiraga F. Enhanced depth imaging spectral-domain optical coherence tomography of subfoveal choroidal thickness in normal Japanese eyes. Japanese journal of ophthalmology 2012;56:230–5. 10.1007/s10384-012-0128-5 22438195

[13] Rossou E, Abegao Pinto L, Vandewalle E, Cassiman C, Willekens K, Stalmans I. Choroidal thickness of the papillomacular region in young healthy individuals. Ophthalmologica Journal international d'ophtalmologie International journal of ophthalmology Zeitschrift fur Augenheilkunde 2014;232:97–101. 10.1159/000360797 24993167

[14] Nishi T, Ueda T, Hasegawa T, Miyata K, Ogata N. Choroidal thickness in children with hyperopic anisometropic amblyopia. The British journal of ophthalmology 2014;98:228–32. 10.1136/bjophthalmol-2013-303938 24187049

[15] Kim M, Kim SS, Kwon HJ, Koh HJ, Lee SC. Association between choroidal thickness and ocular perfusion pressure in young, healthy subjects: enhanced depth imaging optical coherence tomography study. Investigative ophthalmology & visual science 2012;53:7710–7. 23092924 10.1167/iovs.12-10464

[16] Summers JA. The choroid as a sclera growth regulator. Experimental eye research 2013;114:120–7. 10.1016/j.exer.2013.03.008 23528534 PMC3724760

[17] Norton TT, Rada JA. Reduced extracellular matrix in mammalian sclera with induced myopia. Vision research 1995;35:1271–81. 7610587 10.1016/0042-6989(94)00243-f

[18] Nickla DL, Wildsoet C, Wallman J. Compensation for spectacle lenses involves changes in proteoglycan synthesis in both the sclera and choroid. Current eye research 1997;16:320–6. 9134320 10.1076/ceyr.16.4.320.10697

[19] Rada JA, Achen VR, Penugonda S, Schmidt RW, Mount BA. Proteoglycan composition in the human sclera during growth and aging. Investigative ophthalmology & visual science 2000;41:1639–48. 10845580

[20] Woodman-Pieterse EC, Read SA, Collins MJ, Alonso-Caneiro D. Regional Changes in Choroidal Thickness Associated With Accommodation. Investigative ophthalmology & visual science 2015;56:6414–22. 26444722 10.1167/iovs.15-17102

[21] Wallman J, Winawer J. Homeostasis of eye growth and the question of myopia. Neuron 2004;43:447–68. 10.1016/j.neuron.2004.08.008 15312645

[22] Wei WB, Xu L, Jonas JB, Shao L, Du KF, Wang S, et al Subfoveal choroidal thickness: the Beijing Eye Study. Ophthalmology 2013;120:175–80. 10.1016/j.ophtha.2012.07.048 23009895

[23] Kim M, Kim SS, Koh HJ, Lee SC. Choroidal thickness, age, and refractive error in healthy Korean subjects. Optometry and vision science: official publication of the American Academy of Optometry 2014;91:491–6. 24727822 10.1097/OPX.0000000000000229

[24] Margolis R, Spaide RF. A pilot study of enhanced depth imaging optical coherence tomography of the choroid in normal eyes. American journal of ophthalmology 2009;147:811–5. 10.1016/j.ajo.2008.12.008 19232559

[25] Flores-Moreno I, Ruiz-Medrano J, Duker JS, Ruiz-Moreno JM. The relationship between retinal and choroidal thickness and visual acuity in highly myopic eyes. The British journal of ophthalmology 2013;97:1010–3. 10.1136/bjophthalmol-2012-302836 23766433

[26] Sayanagi K, Gomi F, Ikuno Y, Akiba M, Nishida K. Comparison of spectral-domain and high-penetration OCT for observing morphologic changes in age-related macular degeneration and polypoidal choroidal vasculopathy. Graefe's archive for clinical and experimental ophthalmology = Albrecht von Graefes Archiv fur klinische und experimentelle Ophthalmologie 2014;252:3–9. 10.1007/s00417-013-2474-5 24136628

